# Supplementary material for: The cortical connectivity of the periaqueductal gray and the conditioned response to the threat of breathlessness
Source: eLife. 2017 Feb 17;6:e21749. doi: 10.7554/eLife.21749 (PMC5332157; doi:10.7554/eLife.21749)
Supplement: Supplementary file 1. — (A) Supplementary Table 1. Mean (±sd) physiological variables across conditioned respiratory tasks. (B) Supplementary Table 2. Mean (±sd) physiological and psychological variables during breathlessness for both athletes and sedentary subjects. No significant differences were found between groups. DOI: http://dx.doi.org/10.7554/eLife.21749.007 [file elife-21749-supp1.docx]

**Supplementary Table 1.** Mean (±sd) physiological variables across conditioned respiratory tasks. *Significantly (*p* < 0.05) different from unloaded breathing condition. Abbreviations: P_ET_CO_2_, pressure of end-tidal carbon dioxide; P_ET_O_2_, pressure of end-tidal oxygen; RVT, respiratory volume per unit time.

|  | Unloaded breathing | Anticipation of breathlessness | Breathlessness |
| --- | --- | --- | --- |
| Average mouth pressure (cmH_2_O) | -0.35 (0.8) | -0.46 (0.9) | -5.69 (3.0)* |
| Peak mouth pressure (cmH_2_O) | - | - | -14.7 (8.3) |
| P_ET_CO_2_ (mmHg) | 35.5 (4.7) | 35.1 (5.0)* | 35.9 (5.4)* |
| P_ET_O_2_ (mmHg) | 131.9 (11.8) | 131.6 (10.9) | 134.4 (13.0)* |
| Respiratory rate (min^-1^) | 11.8 (3.4) | 11.5 (3.8) | 10.5 (4.5)* |
| RVT (%) | -2.3 (7.0) | 3.0 (15.9)* | -16.6 (26.2)* |

**Supplementary Table 2.** Mean (±sd) physiological and psychological variables during breathlessness for both athletes and sedentary subjects. No significant differences were found between groups.

|  | Athletes | Sedentary |
| --- | --- | --- |
| Peak inspiratory resistance (cmH_2_O) | -14.4 (8.5) | -12.0 (5.8) |
| Breathlessness intensity rating (%) | 46.3 (14.1) | 46.7 (18.1) |
| Breathlessness anxiety rating (%) | 31.9 (17.8) | 36.1 (20.0) |
| Unloaded breathing intensity rating (%) | 2.3 (3.5) | 3.4 (3.4) |
| Unloaded breathing anxiety rating (%) | 2.8 (4.8) | 2.2 (2.7) |
