# Supplementary material for: The cortical connectivity of the periaqueductal gray and the conditioned response to the threat of breathlessness
Source: eLife. 2017 Feb 17;6:e21749. doi: 10.7554/eLife.21749 (PMC5332157; doi:10.7554/eLife.21749)
Supplement: Supplementary file 2. — (A) Supplementary Table 3. Index of data quality and residual head motion using a framewise change in signal intensity. Mean and standard deviation (SD) of DVARS (root mean square of the differential of all timecourses within the mask at each frame) as a percentage of BOLD signal for raw and cleaned data (see below for details on data cleaning procedures). (B) Data preprocessing description. DOI: http://dx.doi.org/10.7554/eLife.21749.008 [file elife-21749-supp2.docx]

**Supplementary Table 3.** Index of data quality and residual head motion using a framewise change in signal intensity. Mean and standard deviation (SD) of DVARS (root mean square of the differential of all timecourses within the mask at each frame, see below) as a percentage of BOLD signal for raw and cleaned data (see below for details on data cleaning procedures).

| Subject | Raw data | | Cleaned data | |
| --- | --- | --- | --- | --- |
|  | MEAN | SD | MEAN | SD |
| 001 | 30.42 | 7.82 | 0.37 | 0.05 |
| 002 | 20.27 | 3.64 | 0.29 | 0.04 |
| 003 | 28.55 | 5.85 | 0.37 | 0.06 |
| 004 | 37.11 | 11.45 | 0.38 | 0.08 |
| 005 | 29.20 | 6.31 | 0.36 | 0.04 |
| 006 | 40.10 | 12.84 | 0.45 | 0.10 |
| 007 | 45.64 | 15.32 | 0.50 | 0.12 |
| 008 | 37.09 | 12.19 | 0.36 | 0.08 |
| 009 | 39.33 | 10.04 | 0.37 | 0.06 |
| 010 | 51.81 | 14.76 | 0.48 | 0.09 |
| 011 | 31.20 | 8.02 | 0.29 | 0.04 |
| 012 | 27.64 | 4.52 | 0.30 | 0.03 |
| 013 | 23.40 | 6.32 | 0.35 | 0.06 |
| 014 | 31.86 | 8.91 | 0.36 | 0.06 |
| 015 | 36.25 | 9.85 | 0.34 | 0.05 |
| 016 | 33.41 | 9.26 | 0.29 | 0.05 |
| 017 | 36.58 | 12.91 | 0.42 | 0.09 |
| 018 | 35.91 | 9.82 | 0.42 | 0.07 |
| 019 | 21.42 | 5.23 | 0.30 | 0.05 |
| 020 | 39.72 | 16.09 | 0.47 | 0.12 |
| 021 | 40.27 | 18.34 | 0.44 | 0.12 |
| 022 | 28.68 | 6.25 | 0.35 | 0.05 |
| 023 | 31.88 | 10.61 | 0.32 | 0.06 |
| 024 | 24.83 | 6.66 | 0.32 | 0.06 |
| 025 | 26.05 | 8.29 | 0.32 | 0.05 |
| 026 | 23.47 | 4.86 | 0.26 | 0.03 |
| 027 | 32.92 | 13.16 | 0.41 | 0.10 |
| 028 | 36.46 | 7.53 | 0.34 | 0.04 |
| 029 | 32.30 | 8.31 | 0.29 | 0.04 |
| 030 | 25.21 | 3.54 | 0.29 | 0.04 |
| 031 | 28.26 | 8.10 | 0.35 | 0.06 |
| 032 | 31.92 | 6.08 | 0.32 | 0.04 |
| 033 | 27.21 | 5.64 | 0.32 | 0.04 |
| 034 | 24.43 | 10.96 | 0.38 | 0.10 |
| 035 | 29.27 | 8.65 | 0.31 | 0.05 |
| 036 | 49.77 | 27.10 | 0.47 | 0.14 |
| 037 | 42.89 | 11.92 | 0.39 | 0.07 |
| 038 | 38.12 | 11.02 | 0.43 | 0.07 |
| 039 | 24.42 | 7.23 | 0.29 | 0.05 |
| 040 | 25.54 | 4.97 | 0.32 | 0.04 |
| MEAN | 32.52 | 9.51 | 0.36 | 0.06 |

**Data preprocessing**

The ‘cleaned data’ described in Supplementary Table 3 is the data following preprocessing and noise correction steps, prior to being fed into the statistical analysis for task or resting state. Image processing was performed using the Oxford Centre for Functional Magnetic Resonance Imaging of the Brain Software Library (FMRIB, Oxford, UK; FSL version 5.0.8; <http://www.fmrib.ox.ac.uk/fsl/)>. Preprocessing steps included:

1. Motion correction and motion parameter recording: using MCFLIRT (Motion Correction using FMRIB's Linear Image Registration Tool) (Jenkinson et al., 2002): https://fsl.fmrib.ox.ac.uk/fsl/fslwiki/MCFLIRT
2. Removal of the non-brain structures (skull and surrounding tissue): using BET (Brain Extraction Tool) (Smith, 2002): https://fsl.fmrib.ox.ac.uk/fsl/fslwiki/BET
3. Spatial smoothing: using a full-width half-maximum Gaussian kernel of 2 mm
4. High-pass temporal filtering: using Gaussian-weighted least-squares straight line fitting (120s)
5. Distortion correction of EPI scans: using a combination of FUGUE (FMRIB's Utility for Geometrically Unwarping EPIs) (Holland et al., 2010; Jenkinson, 2001; Jezzard, 2012) and BBR (Boundary Based Registration; part of the FMRI Expert Analysis Tool, FEAT, version 6.0 (Greve and Fischl, 2009)): http://fsl.fmrib.ox.ac.uk/fsl/fslwiki/FLIRT_BBR
6. Data denoising: using a combination of independent components analysis (ICA) and retrospective image correction (RETROICOR) (Brooks et al., 2013; Harvey et al., 2008), as previously described (Faull et al., 2016b), incorporating head motion regressors.

- ICA: Data was decomposed using automatic dimensionality estimation, and ‘noise’ components (Kelly et al., 2010) as well as head motion regressors (identified previously using MCFLIRT) were removed from the data using linear regression: https://fsl.fmrib.ox.ac.uk/fsl/fslwiki/FIX
- RETROICOR: Physiological recordings of heart rate and respiration from respiratory bellows were transformed into regressors (3 cardiac, 4 respiratory harmonics, an interaction term and a measure of respiratory volume per unit of time (RVT) (Harvey et al., 2008)) corresponding to each acquisition slice, and the signal associated with this noise was isolated using linear regression, adjusted for any interaction with previously-identified ICA noise components and then subtracted from the data: http://fsl.fmrib.ox.ac.uk/fsl/fslwiki/PNM

*Calculation of residual head motion*

There are two common ways to measure residual motion – one is to sum the motion correction parameters, while the other is to calculate intensity differences between realigned volumes (https://fsl.fmrib.ox.ac.uk/fsl/fslwiki/FSLMotionOutliers). The first is typically done using measures such as framewise displacement (an average of rotation and translation parameter differences calculated from motion correction), while the latter can be calculated using measures such as DVARS (D referring to temporal derivative of timecourses, VARS referring to Root Mean Square (RMS) variance over voxels).

Measures such as framewise displacement are dependent on the accuracy of the initial motion correction, and thus may not fully describe the residual motion. Alternatively, a DVARS measure roughly equates to the percentage signal change between volumes, and while this will also incorporate signal changes associated with tasks, it is thought to be more conservative as it is not reliant on the accuracy of the motion correction. We have calculated the mean and standard deviation of the DVARS for our unprocessed data and our final (ICA + RETROICOR) cleaned data, and presented this in Supplementary Table 3. Powers et al. (2012) use a threshold of 0.5% BOLD signal change as an acceptable value for DVARS measurements, and our data shows each subject’s motion as below this threshold following rigorous preprocessing.
